# The Efficiency of Infants' Exploratory Play Is Related to Longer-Term Cognitive Development

**DOI:** 10.3389/fpsyg.2018.00635

**Published:** 2018-05-31

**Authors:** Paul Muentener, Elise Herrig, Laura Schulz

**Affiliations:** ^1^Department of Psychology, Tufts University, Medford, MA, United States; ^2^Department of Brain and Cognitive Sciences, Massachusetts Institute of Technology, Medford, MA, United States

**Keywords:** exploratory play, cognitive development, IQ, infancy, longitudinal design

## Abstract

In this longitudinal study we examined the stability of exploratory play in infancy and its relation to cognitive development in early childhood. We assessed infants' (*N* = 130, mean age at enrollment = 12.02 months, *SD* = 3.5 months; range: 5–19 months) exploratory play four times over 9 months. Exploratory play was indexed by infants' attention to novelty, inductive generalizations, efficiency of exploration, face preferences, and imitative learning. We assessed cognitive development at the fourth visit for the full sample, and again at age three for a subset of the sample (*n* = 38). The only measure that was stable over infancy was the efficiency of exploration. Additionally, infants' efficiency score predicted vocabulary size and distinguished at-risk infants recruited from early intervention sites from those not at risk. Follow-up analyses at age three provided additional evidence for the importance of the efficiency measure: more efficient exploration was correlated with higher IQ scores. These results suggest that the efficiency of infants' exploratory play can be informative about longer-term cognitive development.

## Introduction

Parents, educators, and researchers (Groos, 1901; Vygotsky, 1934/1962; Piaget, 1962; Berlyne, 1969; Bruner et al., 1976; Rubin et al., 1983; Power, 2000) all tend to believe that children learn through exploratory play; however, understanding the relation between play and cognitive development remains an ongoing challenge. The causal relation between exploration and cognitive development has been proposed in both directions: smarter, more behaviorally flexible species are more likely to play (Groos and Baldwin, 1898; Bjorklund, 1997; Pellegrini et al., 2007), and play may support the acquisition of motor (Bjorklund and Brown, 1998; Pellegrini and Smith, 1998), cognitive (Hutt and Bhavani, 1972; Singer et al., 2006), and social skills (Leslie, 1987; Astington and Jenkins, 1995; Youngblade and Dunn, 1995; Taylor and Carlson, 1997) Play has also been used to assess epistemic curiosity, with several studies showing that children selectively engage in exploratory play given opportunities for information gain (Schulz and Bonawitz, 2007; Schulz et al., 2008; Cook et al., 2011; Bonawitz et al., 2012; Buchsbaum et al., 2012; Legare, 2012, 2014; Gopnik and Walker, 2013; Gweon et al., 2014; Stahl and Feigensen, 2015; van Schijndel et al., 2015). Research also suggests that early advances in exploratory play or direct facilitation of exploratory play may have cascading effects on children's learning about the physical and social world (Needham et al., 2002; Sommerville et al., 2005; Libertus and Needham, 2010; Rakison and Krogh, 2012; Schwarzer et al., 2013; Gerson and Woodward, 2014; Oudgenoeg-Paz et al., 2015).

Although a large body of research has attempted to characterize exploratory play over the first few years of life, defining exploratory play remains a challenge. Indeed, although the clinical diagnosis of developmental disorders such as Autism Spectrum Disorders (ASD) and Attention Deficit Hyperactivity Disorder (ADHD) is partly based upon the judgment that children engage in atypical exploratory play (e.g., restricted/repetitive play in ASD, and distracted/disoragnized play in ADHD; American Psychiatric Association, 2013), distinguishing typical and atypical exploratory play remains largely a matter of intuition. Studies that have tried to characterize exploratory play more rigorously have focused largely on how simple object manipulation changes with age. Such studies have assessed, for instance, the number of objects children play with, the amount of time children play with each object, and the types of actions they engage in (e.g., spinning, touching, dropping, banging, etc.,) (McCall, 1974; Fenson et al., 1976; Ruff, 1984; Palmer, 1989; Rochat, 1989; Whyte et al., 1994; Morange-Majoux et al., 1997; Bourgeois et al., 2005; Kahrs et al., 2013). Other work has focused on visual exploration, documenting changes in scan patterns and rate of habituation to novel stimuli over infancy (Fagan, 1974; Rose et al., 1982, 2001; Rose, 1983; Bornstein and Benasich, 1986; Colombo et al., 1987, 1988, 2004; Rose and Feldman, 1987; Richards, 1997). Finally, other work has focused on the relation between visual attention and manual action during object exploration (Fenson et al., 1974; Johnson and Brody, 1977; Ruff, 1986; Ruff and Dubiner, 1987; Oakes et al., 1991, 2002; Ruff et al., 1992; Oakes and Tellinghuisen, 1994; Cassia and Simion, 2002; Perone and Oakes, 2006; Soska et al., 2010; Baumgartner and Oakes, 2013) Such research suggests that children's visual exploration becomes more efficient, (e.g., reflected in faster encoding of visual information), their manual exploration becomes more complex, and the link between their visual and motor systems become more integrated over development. These developments may represent increasingly sophisticated cognitive skills, more opportunities for learning, or both.

It is also the case that relatively few studies have looked at whether individual differences in infants' exploratory play are related to longer-term cognitive development. Rather, studies have looked either at proxy measures, arguably related to but not necessarily specific to exploratory play, or they have looked at single-time-point correlations between measures of exploration and measures of cognition. As a result of such work, we now know, for instance, that one of the most basic measures of visual exploration in infancy—rate of visual habituation—is a better predictor of IQ than standard developmental assessments such as the Bayley's Scales of Infant Development, the Battelle Developmental Inventory, and the Gesell Developmental Assessment (see McCall and Carriger, 1993 for review and meta-analysis). Similarly, a detailed multivariate analysis of 60 min of typically-developing infants' object exploration and overall motor development at 5 months correlates with children's academic achievement at 14 years of age (Bornstein et al., 2013). Additionally infants categorized as high risk for developmental delay (e.g., infants born prematurely, with Down Syndrome, or with older sibling with ASD) differ from full-term infants in their simple object interactions (e.g., touching, rotating, and transferring) (Sigman, 1976; Kopp and Vaughn, 1982; Ruff et al., 1984; Loveland, 1987; Kavsek and Bornstein, 2010; de Almeida Soares et al., 2012; de Campos et al., 2013; Koterba et al., 2014; Kaur et al., 2015; Zuccarini et al., 2016).

Collectively, this research suggests that something about early exploratory play correlates with cognitive development, but which precise aspects of exploratory play are correlated with cognitive development remain unclear. Across studies, researchers have looked variously at discursive vs. focused exploratory play in preschoolers and divergent and convergent thinking in seven to 10-year-olds (Hutt and Bhavani, 1972), stimulation seeking (including, but not limited to, exploratory play) in 3-year-olds and IQ in 11-year-olds (Raine et al., 2002), fine and gross motor development in infancy and literacy at seven (Viholainen et al., 2006), and variables indexing both exploratory activity and motor maturity (upper and lower body coordination, locomotion, and balance) in infants and IQ in adolescence (Bornstein et al., 2013). The diversity of such studies speaks to a compelling relation between early exploration and later cognitive development, but raises questions about whether more active infants are more likely to thrive overall, whether any particular aspects of exploratory play might be particularly informative, and how any particular aspects of exploratory play may be related to each other. In the current study we attempt to address each of these questions. Specifically, using naturalistic measures of exploratory play (i.e., measures that could be easily used in educational, clinical or home environments) we aimed to see (1) whether we could identify diverse, non-overlapping measures of exploration; (2) whether any of these measures were stable longitudinally over infancy, and if so, (3) whether any stable measure of exploratory play correlated with shorter- and longer-term measures of cognitive development.

In choosing which aspects of exploratory play to assess, we were motivated by prior theoretical and empirical work on the role of exploratory play in early cognitive development, and therefore, took a rather broad approach in designing our measures. Our choice of measures was motivated by two overarching perspectives: rational constructivist accounts of children's learning (e.g., Gopnik and Wellman, 2012; Schulz, 2012; Xu and Kushnir, 2013) and social learning theories (Vygotsky, 1934/1962; see also, Tomasello, 2000; Csibra and Gergely, 2006; Meltzoff, 2007). To follow we briefly discuss some of the work underlying the choice of each of the five items in the exploration assessment. Critically, the five items were chosen to be distinctive rather than exhaustive. Our goal was not to fully characterize exploratory play in infancy but to capture components of play that seemed likely to draw on distinct cognitive skills, across different phases of exploratory play (i.e., choosing which objects to explore as well as engaging in different actions on those objects), all while requiring approximately equivalent motor skills (i.e., reaching for and manipulating objects). If exploratory play in early childhood relies upon a single cognitive process, we would expect some or all of these measures of exploratory play to correlate with each other. If, on the other hand, as hypothesized, exploratory play is comprised of a distinct, non-overlapping set of cognitive processes, and our measures effectively assess this, then there should be no correlations among our diverse measures of exploratory play.

Rational constructivist theories propose that at least in simple contexts, children integrate prior knowledge and data to guide their inferences in ways that can be characterized by formal accounts of learning (Tenenbaum et al., 2011). These accounts view children's exploration as an effective means of gathering evidence to inform and update learners' beliefs about the world (see Schulz, 2012 for discussion and review). Here we focus on three aspects of rational exploration: attention to novelty, inductive generalization, and efficiency of exploration.

As noted, infants' attention to novelty has been shown to be one of the most robust predictors of cognitive development: studies of visual attention have shown that faster rates of visual habituation (e.g., fewer trials to reach a habituation criterion, greater decrement in looking time across habituation trials) as well as a greater degree of novelty preference (e.g., longer looking at novel images compared to familiar images) exhibited during looking time studies is correlated with higher IQ and distinguishes full-term from pre-term infants at risk for developmental delay (for review, see McCall and Carriger, 1993; Kavšek, 2004; Fagan et al., 2007) These studies support the argument that encoding and storing visual information more quickly into memory might allow for more opportunities both to integrate this information with existing knowledge and more opportunities to encode new information. To the extent that these measures index visual *exploration*, these findings provide support for the hypothesis that early measures of exploration might index broader cognitive abilities. Because here we were interested in play *per se*, we used manual exploration rather than looking time to assess children's attention to novelty.

The inductive generalization measure was motivated similarly by rational constructivist approaches to early learning. Research suggests that infants can draw rich generalizations from sparse data (Dewar and Xu, 2010; Gweon et al., 2010; Téglás et al., 2011) and that the ability to make inductive generalizations supports much of children's theory-building over the first several years of life (for review, see Schulz, 2012). Thus, it seemed likely that children's ability to make inductive generalizations may be positively related to cognitive development. Here we assessed infants' ability to extend non-obvious properties demonstrated on a target toy to a novel object that had a similar shape, but different color or pattern (e.g., Baldwin et al., 1993; Welder and Graham, 2001).

The efficiency of exploration measure was motivated by work looking at the increasing sophistication of exploratory play over infancy (e.g., Ruff et al., 1992) and the idea that this might play a role in rational exploration (e.g., Bonawitz et al., 2011; Gopnik and Walker, 2013; Legare, 2014; Stahl and Feigensen, 2015; van Schijndel et al., 2015; Sim and Xu, 2017) Further support for this measure comes from some longitudinal work, mentioned above, suggesting that a factor combining both motor coordination and efficient exploratory behavior in infancy correlates with longer-term cognitive development (Bornstein et al., 2013). In the current study, efficient exploration was indexed by the ability to find different target functions on a multi-function toy.

In addition to these three measures focused on rational constructivist learning, we also included two measures intended to assess social aspects of early exploration. First, motivated by considerable evidence that selective attention to faces and face-like stimuli emerges early (for review, Morton and Johnson, 1991; Johnson et al., 2015; see also, Fantz, 1963; Farroni et al., 2002; Johnson, 2005; Frank et al., 2009, 2012; Reid et al., 2017), we thought it was possible that such selective attention might encourage selective exploration. Previous work on exploratory play has focused almost exclusively on object exploration, however, it seemed possible that selective exploration of faces might correlate with later cognitive development. Thus, as we were interested in exploratory play, we assessed infants' preferential exploration of stimuli with faces over stimuli without faces in a reaching task, rather than a traditional preferential looking task.

The second social aspect we assessed was children's imitative learning. We reasoned that although infants' exploratory play is typically assessed as spontaneous, self-directed exploration, in the cultures in which these assessments typically occur, caregivers routinely use ostensive, pedagogical cues to demonstrate object properties to children. Researchers have suggested that infants' responsiveness to pedagogical cuing plays a critical role in cultural transmission (Tomasello, 2000; Csibra and Gergely, 2006) and empirical evidence suggests that the presence or absence of such social cuing changes the way children explore their environment (Senju and Csibra, 2008; Bonawitz et al., 2011; Butler and Markman, 2014; Gweon et al., 2014; Butler and Tomasello, 2016; Shneidman et al., 2016). Motivated by the idea that the ability to use these cues to filter out distractors and constrain initial exploration might be an important cue to cognitive development, we assessed children's imitation of an object function from an adult's pedagogical demonstration.

Thus, to address our first two aims, we assessed the distinctiveness and stability of children's performance on five aspects of exploratory play: attention to novelty, inductive generalization, efficiency of exploration, face preferences, and imitative learning. To capture a broad and representative view of exploratory play over development, we assessed infants' exploratory play over a relatively large age range (5–19 months of age) and across differing levels of risk status for developmental delay (i.e., a subset of infants were recruited from early intervention sites). In total, throughout the first phase our study (Phase 1), we assessed children's performance on the five exploratory play tasks four times over a 9-month period.

Given that researchers have theorized that the five aspects of exploratory play measured in the current study contribute to learning over the first few years of life, we hypothesized that children's performance on the exploratory play measures might also be indicative of longer-term cognitive development and intelligence. To address this third aim, we assessed the relation between children's exploratory play behaviors and their cognitive development at two time points: in the shorter term at the end of Phase 1 (shorter-term cognitive development assessments described below in Methods) and in the longer-term at 3 years of age (Phase 2 described below in Methods). We specifically looked only at those exploratory play behaviors that were stable over Phase 1. Of course, we anticipated that significant differences would emerge across development at any given time point (e.g., we might expect older children to engage in more efficient exploration than younger children) as well as within participants across Phase 1 (e.g., we might expect children to become more efficient in their exploration over time). Thus, rather than compare children's actual exploratory behavior on each task with other cognitive measures, we looked at how each child performed relative to similar-aged peers at each time-point; although significant developmental changes were likely to occur in our battery of tasks, assessing individual children's abilities relative to their peers should normalize any group-level developmental differences. We reasoned that if children's exploration relative to their peers at one time point failed to predict their exploration relative to their peers at another time point, it was also unlikely to correlate with broader cognitive development. However, to the degree that any measures of exploratory behavior remained stable relative to peers over development, we might then ask how exploratory play correlates both with shorter-term measures of cognitive development and whether exploratory play in infancy correlates with cognitive outcomes later in childhood.

In choosing measures of cognitive development, we focused on broad cognitive abilities that seemed likely to index overall learning and knowledge construction. Specifically, for shorter-term cognitive development we focused on vocabulary size and the ability to delay gratification. Both receptive and productive language abilities contribute to IQ tests, such as the Weschler Preschool and Primary Scales of Intelligence (WPPSI) test (Wechsler, 2012), and vocabulary size in infancy and toddlerhood is correlated with later IQ (Bornstein, 1985; Marchman and Fernald, 2008). Several researchers have also argued that the development of executive function plays a role in conceptual change and theory development across childhood (Carlson and Moses, 2001; Carey et al., 2015; Powell and Carey, 2017) Specifically, within the set of abilities that comprise executive functions (e.g., inhibition, set shifting, working memory), we focused on the ability to delay gratification in early childhood as it has been shown to be correlated with higher IQ later in development (Mischel et al., 1989; Shoda et al., 1990). For the longer-term cognitive development measures, in addition to measuring their IQ and ability to delay gratification, we also included an assessment of children's social communication abilities as we had also focused on social aspects of exploratory play.

To summarize, we assessed the stability and distinctiveness of five aspects of exploratory play in infancy, as well as their potential relation to shorter- and longer-term cognitive development. The study had two phases: in Phase 1 (Exploratory Play Assessment and Shorter-term Cognitive Development Assessment), we assessed infants' exploratory play four times over a 9-month period and, in Phase 2 (Longer-term Cognitive Development Assessment), these children returned for follow-up cognitive assessments at age three. Our overall hypothesis was that components of exploratory play in infancy would be related to cognitive development later in childhood; however, since there is broad agreement among researchers that the individual components tested here may be important for early learning but little consensus as to their relative importance, we remained agnostic as to which specific components of infants' exploratory play would correlate with cognitive development. Phase 1 allowed us to assess the independence of the exploratory tasks from each other, their stability across testing sessions, and their sensitivity to group differences in at-risk vs. typically developing infants. As an exploratory measure, it also allowed us to investigate possible correlations between items on the exploratory play assessment and shorter-term cognitive development in order to motivate a targeted hypothesis for Phase 2. Following these exploratory analyses, we then restricted our analyses of the longer-term relations between exploratory play and cognitive development to the specific components of early exploration that were correlated with shorter-term measures of cognitive development in Phase 1. In order to draw conclusions on the overall relation between exploratory play and cognitive development, we then assessed the relation these components and both the average performance across Phase 1 as well as performance for the first Phase 1 visit.

## Methods

### Participants

We recruited infants between 5 and 19 months of age to participate in this longitudinal study of exploratory play. To increase variability in the sample, we recruited both infants from a local children's museum and infants in early intervention programs. We refer to the former subset of infants as “typically-developing” as these infants were not born premature, were not enrolled in early intervention programs, and had parents who did not report any health concerns for them. We refer to the latter subset of infants as “at-risk,” as these children were enrolled in early intervention services due to birth complications and social risk factors and were expected to be at an increased risk for developmental delay.

For the typically-developing sample, 262 infants were initially recruited at a local children's museum and asked to participate in Visit 1 of the exploratory play assessment (i.e., the first session of this longitudinal study; full procedure described below). At the conclusion of this session, all families were asked if they were interested in continuing on in the remainder of the longitudinal study. Of these 262 infants, 196 (74.81%) families agreed to be contacted for subsequent visits; however, only 120 infants (45.80 %) were scheduled and participated past Visit 1. These 120 infants were contacted every 3 months to participate in Visits 2-4 of Phase 1 of the study. Infants needed to complete at least 3 of the 4 Phase 1 visits in order to be included in the final sample; 96 infants (80.00%) met this criterion, while the remaining infants had families who moved during Phase 1 (*n* = 7), were no longer interested in participating after Visit 2 (*n* = 4), or expressed interest in participating but were unable to schedule 3 or more visits (*n* = 13).

For the at-risk sample, infants were recruited for participation from early intervention programs. Infants had been referred to the early intervention programs due to a combination of risk factors including: prematurity, low birth weight, birth complications, and social risk factors (in particular, low socio-economic status and risk for maternal depression). Contacted families were concurrently enrolled in a separate study assessing maternal problem-solving strategies. Forty-two infants were recruited initially; 38 (90.48%) were scheduled and participated past Visit 1. Of these 38 infants, 34 infants (89.47%) were assessed at three of the four Phase 1 visits; the remaining infants had families who moved during Phase 1 (*n* = 1), were no longer interested in participating after Visit 2 (*n* = 1), or were interested in participating but unable to schedule 3 or more visits (*n* = 2).

Thus, the final sample of participants who participated in at least three of four Phase 1 visits over the 9-month period included 130 children (69 female): 96 typically developing infants (*n* = 51 female) and 34 at-risk infants (*n* = 18 female) (overall mean age at enrollment: 12.02 months, *SD* = 3.5 months; range: 5–19 months).

Families were contacted again when their child turned three to participate in Phase 2 of the study. All follow-up visits were completed within approximately 6 months of the child's third birthday. Of the initial sample of 130 infants, 38 children returned for Phase 2 (29.23%; mean age at Phase 2 assessment: 3.23 years, *SD* = 0.15 years; range 36–43 months); two of these children were from the at-risk sample.

### Procedure

**Figure 1:**
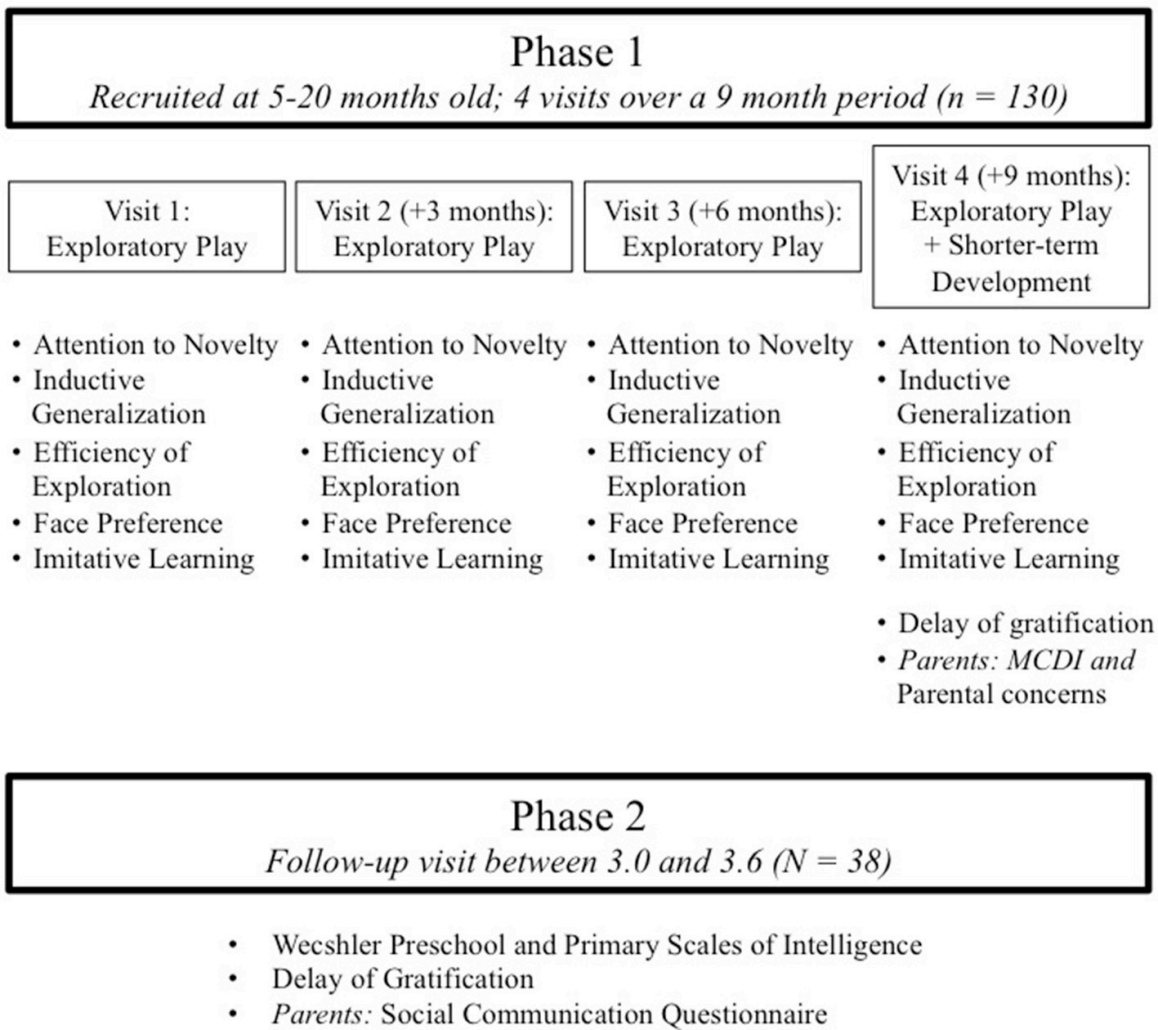
Description of the overall study design.

The study has two phases: the Exploratory Play Assessment and Shorter-term Cognitive Development Assessments (Phase 1) and Longer-term Cognitive Development Assessment (Phase 2). See Figure 1 for study design. All procedures were approved by the MIT Institutional Review Board with written informed consent provided by the parents of all participants in this study.

In Phase 1 of the study (Figure 1), we administered an exploratory play assessment to infants four times over a 9-month period. Children began Phase 1 when they were 5–19 months of age and ended Phase 1 when they were 14–28 months of age. After the exploratory play assessment was administered at the final (fourth) Phase 1 visit, parents were asked to complete the Macarthur-Bates Communicative Development Inventory (MCDI; Fenson et al., 2000). To assess the specificity of any significant relation between exploratory play and shorter-term cognitive development, children's executive function skills were assessed on a modified delay of gratification task, and parents were asked to fill out a questionnaire relating to assessment and diagnosis of developmental disorders as well as parental concern.

Children returned for Phase 2 of the study at 3 years of age, at which time an independent lab, with no knowledge of the children's performance on the exploratory play assessment, assessed the children's IQ using the Weschler Preschool and Primary Scales of Intelligence (WPPSI) test (Wechsler, 2012). To determine the specificity of any relation between exploratory play and IQ, the children's executive functioning (Mischel et al., 1989) and social communication abilities (Rutter et al., 2003) were also assessed.

#### Phase 1: exploratory play assessment

**Figure 2:**
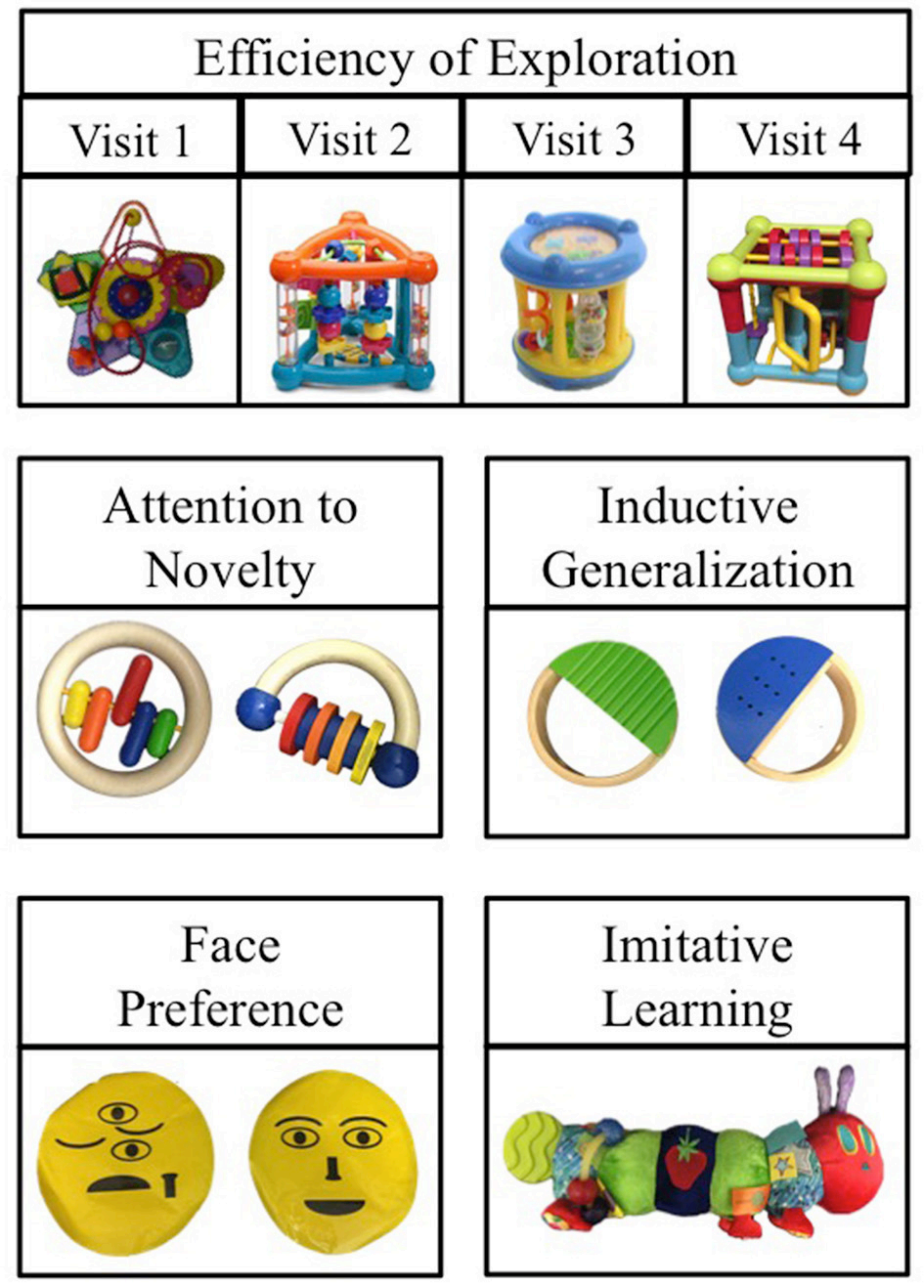
Sample stimuli images. All four Efficiency stimuli are shown below. Sample stimuli from the remaining tasks are shown below; see Table 1 for a description of the full stimulus set.

The exploratory play assessment took approximately 15 min to complete. Infants were tested in a quiet room in their own homes, a private testing room in our laboratory, or an onsite laboratory at a children's museum; a preliminary assessment early in the data collection process showed that the procedure could be implemented equally well across testing locations. Parents were present throughout the procedure, but were not told any of the dependent measures or directional hypotheses for any task or for the study overall. A striped red tablecloth was placed between the experimenter and the child in order to control for stimuli placement throughout the study. The procedure described below was the same at each of the four Phase 1 visits; however, we used different stimuli at each visit (see Figure 2 for example stimuli; see Table 1 for full details). The same experimenter administered the exploratory play assessment at each visit across Phase 1. All sessions were videotaped and all behaviors were coded from videotape. Although this experimenter was present across all Phase 1 visits, the experimenter did not code children's performance on these tasks and did not view coded data for individual children when conducting Phase 1 visits.

**Table 1:** Materials used in the exploratory play assessment.

**Task**	**# Stimuli**	**Items**	**Target functions**
Attention to novelty	2 pairs/visit (16 total)	Multi-colored rattles, plush balls, multi-colored objects	n/a
Inductive generalization	2 pairs (1 functioning, 1 inert)/visit (16 total)	Visit 1: Rattle; castanet Visit 2: Rattle; magnet Visit 3: Bell; light-up wand Visit 4: Tube; blocks	Visit 1: rattle noise; clicking noise Visit 2: rattle noise; sticking together Visit 3: ringing noise; lighting up Visit 4: whirly noise; music
Efficiency of exploration	1 novel toy/visit (4 total)	Visit 1: Star-shaped toy Visit 2: Triangle-shaped toy Visit 3: Cylinder toy Visit 4: Cube toy	Visit 1: moveable beads, spinning ball, squeaking button, crinkly fabric, central button, underside of toy Visit 2: moveable balls, rotating disc, moveable beads, moveable disc, rattle beads Visit 3: moveable rings, stars, discs, spinning beads, underside of toy, and rotating disc Visit 4: mirror, spinning beads, moveable disc, spinning shapes, moveable beads, rotating disc
Face preference	3 pairs/visit (24 total)	Schematic, upright face with line-drawn features Scrambled face with same features randomly placed	n/a
Imitative learning	2 toys/visit (8 total)	Visit 1: Plush toy; 3-tiered toy Visit 2: Flower toy; plush ball Visit 3: Fish toy; round, plush toy Visit 4: Spiral toy; plush ball	Visit 1: squeaking center when pressed; spinning middle tier Visit 2: spinning discs on stem; pulled apart into 4 wedges Visit 3: wiggling fin; turning over and squeaking red button Visit 4: pulling tab to vibrate; pulling out bear from center

##### Warm-up phase

This trial helped familiarize the children to the experimenter and determine the extent of each child's furthest reach. During this phase, the experimenter established the child's furthest reach to the left, right, and center of the tablecloth with a toy not in the stimulus set. When children had to make a choice between stimuli during Phase 1, the experimenter placed the items at the limits of each child's reach.

##### Attention to novelty task

We assessed children's exploration of novel toys on two trials (Fenson et al., 1974; Sigman, 1976; Oakes et al., 1991, 2002) At the start of each trial, the experimenter said, “Look at this!” while holding up a toy (familiar toy). The experimenter then placed the familiar toy within the child's reach and allowed the child to play for 30 s. The experimenter then retrieved the familiar toy and showed the child the familiar toy alongside a new toy (novel toy). The researcher then placed both toys equidistant to the left and right of the child (counterbalanced across children) and allowed the child to play for up to 90 s. The experimenter then repeated this procedure with a new pair of stimuli on the second trial. We coded the child's latency to touch the novel toy on each trial and averaged the latencies to compute an average latency.

##### Efficiency of exploration task

We assessed how long children explored a novel multi-function toy on a single trial and how many functions of the toy they contacted (adapted from Bonawitz et al., 2011; Gweon et al., 2014; Shneidman et al., 2016). At the start of the trial, the experimenter said, “Look at this!”, placed the toy within the child's reach and allowed them play. The play time was terminated when any of the following occurred: (1) the child stopped contacting the toy for 5 s, the toy was re-introduced to the child, and the child again stopped contacting the toy for 5 s; (2) the child verbally indicated that they were finished or (3) 5 min of play time elapsed, whichever came first. The different functions for each toy were pre-specified based on the individual toys. We coded the total time the child was in contact with the toy as well as the number of pre-specified functions of the toy the child discovered. We divided the number of functions the child found by the total amount of time the child played with the toy to yield an efficiency score. Note that because this measure does not compensate for the fact that later-discovered functions may be more difficult to find, it is a relatively conservative measure of the efficiency of children's exploration.

##### Inductive generalization task

We tested children's ability to generalize non-obvious properties of objects (Baldwin et al., 1993; Welder and Graham, 2001). At the start of each trial, the experimenter said, “Look at this!” while holding up a novel toy. She then demonstrated a target action on the toy (e.g., shaking it to make a rattle noise) six times. The experimenter then gave the child a new toy that was the same shape but differed in color and pattern. The child's toy was inert (e.g., it did not make a noise when shaken). The child was allowed to play for up to 30 s, and we coded the number of target actions the child produced. The experimenter repeated this procedure on a second trial with new toys and outcomes. During the second trial, the child's toy produced the target outcome so that the child could not infer that the toys would never produce the target outcome. The experimenter then repeated the procedure on a third trial, again with new toys and outcomes; as in the first trial, the child's toy did not produce the target outcome. We averaged the number of target actions the child produced on the first and third trial to yield the average number of attempts.

##### Face preference task

We assessed whether children preferred toys with schematic upright faces to schematic scrambled faces using a forced choice paradigm (adapted from Morton and Johnson, 1991). At the start of each trial the experimenter said, “Look at this one!” while holding up a schematic face and then a scrambled face, both mounted on discs. The experimenter then placed the discs equidistant to the left and right of the child (counterbalanced across children) and allowed them to make a choice. This procedure was repeated twice more with new stimuli. We coded whether the child chose the face on each trial yielding a % preference for face stimuli.

##### Imitative learning task

We assessed the extent to which children would imitate a pedagogically demonstrated target action (e.g., Southgate et al., 2009). Pilot testing on each toy was used to identify children's initial actions at baseline (e.g., playing with feet and antennae of plush caterpillar toy); the experimenter's target actions were always actions never produced by children at baseline. At the start of each trial, the experimenter said, “Look at my toy! This is my toy. I am going to show you how my toy works. Watch!” and then demonstrated a target action (e.g., pushing center of caterpillar toy to make a squeaking noise). The experimenter then said, “Wow! That's how my toy works. Watch, this is how my toy works,” and demonstrated the same target action two additional times. The experimenter then said, “Do you want to play with my toy?” and placed the toy within the child's reach. We coded whether the child imitated the experimenter's action on the first interaction with the toy (1 or 0). This procedure was repeated on a second trial with a new toy. We summed across the two trials to yield a total imitation score.

#### Phase 1: shorter-term cognitive development assessment

We assessed children' vocabulary and executive function abilities as well as asked parents about any developmental concerns as a measure of shorter-term cognitive development outcomes. These assessments occurred at the final (fourth) Phase 1 visit, when children were between 14 and 28 months of age. For two participants, the vocabulary measure and parent questionnaire were completed over the phone, as the participants did not complete a fourth visit; these participants did not provide data for the delay of gratification task.

##### Vocabulary

To assess children's vocabulary size, parents completed the short form Macarthur-Bates Communicative Development Inventory (MCDI), which assesses children's receptive and productive vocabulary (Fenson et al., 2000). This inventory was then scored corresponding to the child's corrected-age based on prematurity. Children whose corrected age was under 18 months were assessed using the CDI: Words and Gestures form; children whose corrected age was over 18 months were assessed using the CDI: Words and Sentences form. We determined children's percentile score based on the productive vocabulary measure across both forms.

##### Delay of gratification task

Children were shown that when a ball was placed down a chute, a jingle noise would occur. Children were very interested in this outcome, and most children spontaneously reached for the ball to place it down the chute. The experimenter, however, kept the ball and chute at a distance from the child. The experimenter then placed the ball under a transparent cup, and children were told that they needed to wait to retrieve the ball until the experimenter rang a bell. The experimenter increased the wait time on successive trials (5, 10, 20, 40, and 80 s), and we averaged the time it took for children to retrieve the ball across trials.

##### Parental concerns checklist

Parents reported whether their child had ever spent time in a neonatal intensive care unit, had ever been assessed for any developmental disorder, and whether they had any concerns about their child's motor, social, language, or cognitive development. Children who spent time in the neonatal intensive care unit or whose parents reported any concern about their development were given a score of 1; all other children were given a score of 0.

#### Phase 1: administration and coding

**Figure 3:**
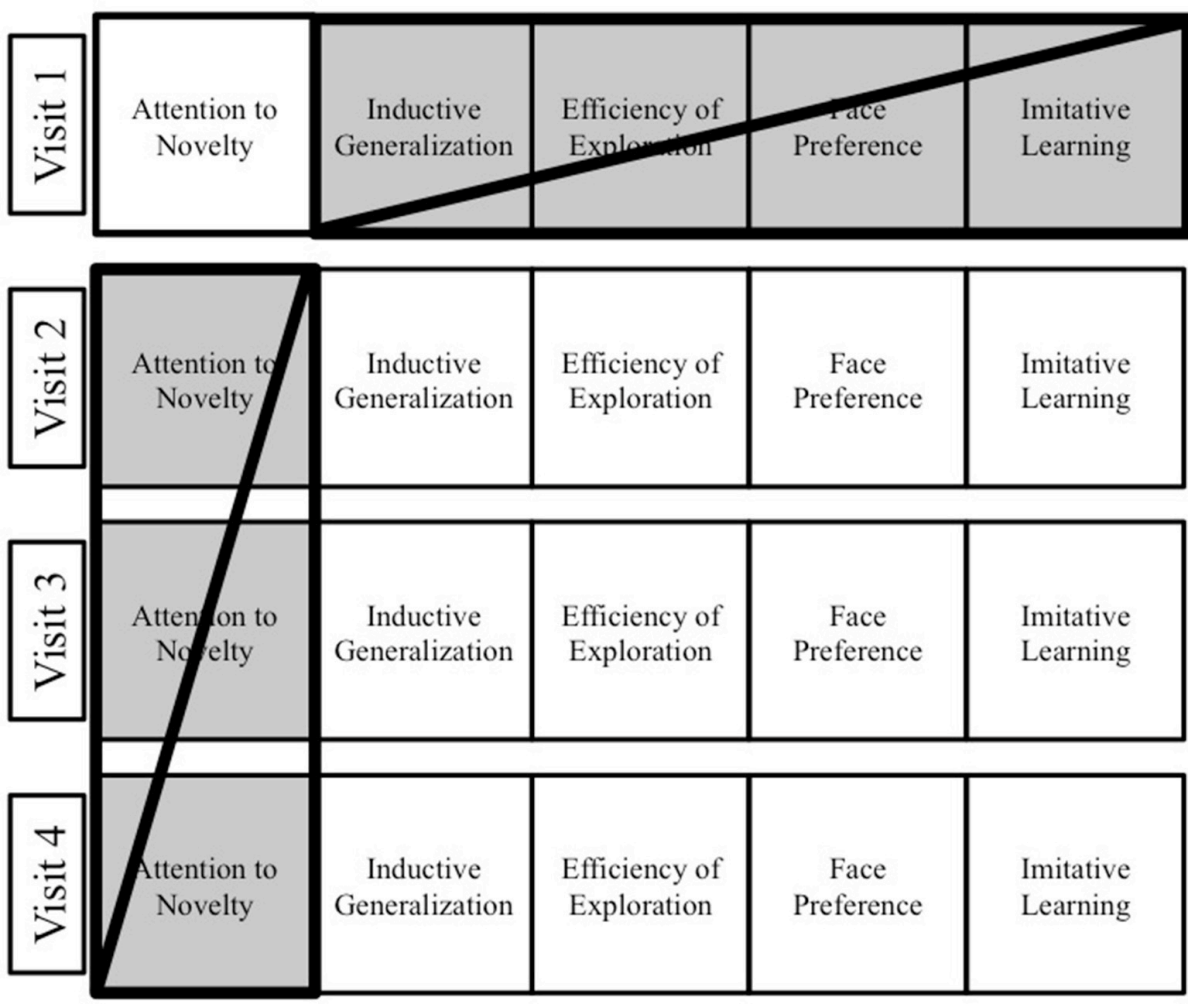
Visual depiction of coding procedure. Coders coded no more than one task within a visit and no more than one visit for a given task. For example, if a coder coded the Visit 1 Attention to Novelty task for a participant, then that coder did not code any other Visit 1 task or the Attention to Novelty task on any other visit for that participant.

A single experimenter administered the exploratory play assessment throughout Phase 1. This experimenter neither coded nor saw any of the Phase 1 data. Eighteen different coders independently coded the videotapes from the Phase 1 exploratory play assessment. The coders were unaware of Phase 2 and that some children were at-risk for developmental delay, and did not know the directional hypotheses for any task or the overall study. To mitigate against any bias from coding repeated tasks for a given child, the coders' responsibilities were distributed such that any given coder coded only one of the five tasks in a single visit and did not code the same task across visits (e.g., a coder who coded the Visit 1 Attention to Novelty task did not code this child on any other Visit 1 task and did not code the Visits 2-4 Attention to Novelty task for that child) (Figure 3). All coders were initially trained to code performance on all five exploratory play tasks in this study, using testing sessions from children (*n* = 20) who had completed only the first Phase 1 visit. All coders achieved high inter-rater reliability (all *r's* >0.9) with experienced coders on each of the five exploratory play tasks. An additional two coders coded the delay of gratification task; both were unaware of the whether the children were at-risk for developmental delay and had no knowledge of children's Phase 1 performance.

#### Phase 2: longer-term cognitive development assessment

We contacted families for a follow-up visit within 6 months of the child's third birthday. A researcher from an independent clinical lab not involved in any of the previous research, unaware of children's risk status, and of children's performance in Phase 1, administered the Phase 2 assessments: the IQ test and delay of gratification task. Parents completed the Social Communication Questionnaire (SCQ) (Rutter et al., 2003) while the children were completing the other tasks. The independent researcher coded all tasks.

##### IQ task

We assessed IQ at age 3 with the Weschler Preschool and Primary Scales of Intelligence test (WPPSI, 4th edition). This test assessed children's receptive and productive vocabulary, their general world knowledge, and their visual-spatial abilities. We used the full-scale composite score comprised from the individual subscales of the WPPSI as an index of children's cognitive development; we also conducted *post-hoc* analyses using the individual WPPSI verbal comprehension, visual spatial, and working memory subscales.

##### Delay of gratification task

This task was modeled after the standard marshmallow delay of gratification task (Mischel et al., 1989). Children first practiced ringing a bell to make an experimenter return to the room after leaving. Children were left alone in the testing room with a small amount of a preferred snack and told that they could ring the bell immediately to have the small snack or wait until the experimenter returned (without ringing the bell) to have a larger amount of snack. Children were left alone in the testing room for up to 15 min, until they rang the bell, or requested that the experimenter return.

##### Social communication abilities

While the children were completing these tasks, parents completed the Social Communication Questionnaire (SCQ) (Rutter et al., 2003). This questionnaire assesses children's basic social communication abilities (e.g., emotional expressions, turn-taking, pretend play). Although this checklist questionnaire was designed primarily as a screening tool to assist in the diagnosis of autism spectrum disorders in children aged 4 years and older, it has been used successfully to screen for social communication abilities more broadly at 3 years of age (Allen et al., 2007; Snow and Lecavalier, 2008). For diagnostic purposes, the SCQ has a cutoff point of 15 for children older than 4 years of age; a lower cutoff point (e.g., 13) has been recommended for younger children (Snow and Lecavalier, 2008). In the current study we used children's raw score as a continuous measure of their social communicative abilities; however, as we also note below, no child received a score greater than the diagnostic cut-off of 13 on this measure.

## Results

### Preliminary analyses

Preliminary analyses revealed that the Attention to Novelty, Inductive Generalization, Efficiency of Exploration, and Imitative Learning tasks, as well the Delay of Gratification scores during the shorter-term cognitive development assessment, were all correlated with age: performance increased with age for each task (all *ps* < 0.05). Since we were primarily interested in individual differences, rather than age-related differences, participants were split into 3-month cohorts based on their age at enrollment (6-month-old cohort, range: 5–7 months, *n* = 21; 9-month-old cohort, range: 8–10 months, *n* = 35; 12-month-old cohort, range: 11–13 months, *n* = 35; 15-month-old cohort, range: 14–16 months, *n* = 27; 18-month-old cohort, range: 17–19 months, *n* = 12) and a standard score for infants' performance on each task was computed, relative to children in their age cohort, separately for each visit; premature infants were assigned to cohorts based on their age corrected for prematurity. We then computed the average of the standard scores across visits for each task to obtain a measure of infants' average performance on each task relative to similar-aged peers. Subsequent correlational analyses on the average standard scores of each task with participant age, separately by cohort (i.e., 5 task analyses per cohort, 5 cohorts in total), did not reveal any systematic relations and suggested that the new age cohorts mitigated any age effects present in the exploratory play data. Tables 2–7 report the descriptive statistics for all of the raw data for each task, separately by age cohort and visit, as well as the shorter- and longer-term cognitive development measures. These tables show that children's performance resulted in a wide range of raw scores, and suggest that we had sufficient variability to detect potential relations between the measures in the current study.

**Table 2:** Descriptive statistics for the 6-month-old cohort's performance on the Phase 1 Exploratory Play Tasks.

	**Visit #**	**# of Participants**	***M***	***SD***	**Range: Minimum–maximum**
**6-MONTH-OLD COHORT**					
Attention to novelty	Visit 1	20	22.39	14.18	1.67–54.09
	Visit 2	18	14.87	15.94	0–56.40
	Visit 3	17	4.82	8.31	0–30.67
	Visit 4	12	2.83	5.04	0–16.74
Inductive generalization	Visit 1	21	1.26	1.67	0–5.5
	Visit 2	17	2.82	2.65	0–8.5
	Visit 3	16	2.50	2.67	0–9.5
	Visit 4	13	5.00	4.53	0–15
Efficiency of exploration	Visit 1	21	0.021	0.01	0.01–0.04
	Visit 2	18	0.024	0.02	0.01–0.07
	Visit 3	17	0.031	0.02	0.01–0.06
	Visit 4	17	0.035	0.02	0.01–0.1
Face preferences	Visit 1	20	0.61	0.29	0–1
	Visit 2	18	0.41	0.27	0–1
	Visit 3	18	0.50	0.21	0–0.67
	Visit 4	16	0.51	0.29	0–1
Imitative learning	Visit 1	19	0.32	0.34	0–1
	Visit 2	18	0.44	0.42	0–1
	Visit 3	17	0.56	0.39	0–1
	Visit 4	16	0.78	0.26	0.5–1

*Visits occurred at 3-month-intervals across Phase 1: infants were 6, 9, 12, and 15 months of age for Visits 1–4, respectively*.

**Table 3:** Descriptive statistics for the 9-month-old cohort's performance on the Phase 1 Exploratory Play Tasks.

	**Visit #**	**# of Participants**	***M***	***SD***	**Range: Minimum–maximum**
**9-MONTH-OLD COHORT**					
Attention to novelty	Visit 1	35	15.17	19.30	0.52–60
	Visit 2	28	6.87	12.97	0–60
	Visit 3	30	3.21	6.16	0–31.1
	Visit 4	28	3.52	6.76	0–31.25
Inductive generalization	Visit 1	34	3.28	4.40	0–16.5
	Visit 2	30	4.85	4.47	0–18
	Visit 3	28	6.5	5.20	0–25
	Visit 4	26	7.17	5.37	0.5–24.5
Efficiency of exploration	Visit 1	34	0.05	0.04	0.01–0.18
	Visit 2	27	0.03	0.02	0.01–0.09
	Visit 3	29	0.04	0.03	0.01–0.13
	Visit 4	33	0.04	0.04	0.01–0.21
Face preferences	Visit 1	35	0.48	0.24	0–1
	Visit 2	28	0.54	0.29	0–1
	Visit 3	26	0.55	0.28	0–1
	Visit 4	30	0.61	0.33	0–1
Imitative learning	Visit 1	28	0.44	0.36	0–1
	Visit 2	26	0.46	0.30	0–1
	Visit 3	34	0.60	0.36	0–1
	Visit 4	28	0.82	0.33	0–1

*Visits occurred at 3-month-intervals across Phase 1: infants were 9, 12, 15, and 18 months of age for Visits 1-4, respectively*.

**Table 4:** Descriptive statistics for the 12-month-old cohort's performance on the Phase 1 Exploratory Play Tasks.

	**Visit #**	**# of Participants**	***M***	***SD***	**Range: Minimum–maximum**
**12-MONTH-OLD COHORT**					
Attention to novelty	Visit 1	35	6.42	11.14	0.27–43.22
	Visit 2	30	4.01	5.36	0–22.47
	Visit 3	27	3.81	7.87	0–30.2
	Visit 4	31	2.33	5.61	0–31.08
Inductive generalization	Visit 1	34	4.91	4.03	0–15.5
	Visit 2	30	4.88	3.75	0–15.5
	Visit 3	28	5.41	5.29	0–23
	Visit 4	31	8.10	7.27	2–34
Efficiency of exploration	Visit 1	35	0.06	0.05	0.02–0.19
	Visit 2	30	0.04	0.03	0–0.19
	Visit 3	28	0.05	0.05	0.01–0.27
	Visit 4	34	0.04	0.04	
Face preferences	Visit 1	35	0.54	0.29	0–1
	Visit 2	30	0.53	0.27	0–1
	Visit 3	27	0.55	2.6	0–1
	Visit 4	33	0.47	0.32	0–1
Imitative learning	Visit 1	35	0.60	0.36	0–1
	Visit 2	30	0.65	0.35	0–1
	Visit 3	28	0.66	0.33	0–1
	Visit 4	33	0.89	0.24	0–1

*Visits occurred at 3-month-intervals across Phase 1: infants were 12, 15, 18, and 21 months of age for Visits 1-4, respectively*.

**Table 5:** Descriptive statistics for the 15-month-old cohort's performance on the Phase 1 Exploratory Play Tasks.

	**Visit #**	**# of Participants**	***M***	***SD***	**Range: Minimum–maximum**
**15-MONTH-OLD COHORT**					
Attention to novelty	Visit 1	26	4.53	7.46	0–29.95
	Visit 2	21	4.07	8.79	0–30.32
	Visit 3	24	1.78	3.32	0–15.35
	Visit 4	22	1.39	2.23	0–10.85
Inductive generalization	Visit 1	27	7.35	5.62	0.5–25.5
	Visit 2	19	5.76	4.11	0–17
	Visit 3	23	6.98	5.15	0–17.5
	Visit 4	23	9.33	7.12	1.5–30
Efficiency of exploration	Visit 1	27	0.09	0.08	0.02–0.37
	Visit 2	20	0.03	0.02	0.01–0.11
	Visit 3	23	0.05	0.03	0.01–0.15
	Visit 4	23	0.03	0.02	0–0.09
Face preferences	Visit 1	26	0.57	0.27	0–1
	Visit 2	21	0.45	0.26	0–1
	Visit 3	24	0.53	0.31	0–1
	Visit 4	19	0.49	0.25	0–1
Imitative learning	Visit 1	27	0.74	0.32	0–1
	Visit 2	21	0.62	0.35	0–1
	Visit 3	22	0.89	0.21	0.5–1
	Visit 4	23	0.78	0.29	0–1

*Visits occurred at 3-month-intervals across Phase 1: infants were 15, 18, 21, and 24 months of age for Visits 1-4, respectively*.

**Table 6:** Descriptive statistics for the 18-month-old cohort's performance on the Phase 1 Exploratory Play Tasks.

	**Visit #**	**# of Participants**	***M***	***SD***	**Range: Minimum–maximum**
**18-MONTH-OLD COHORT**					
Attention to novelty	Visit 1	11	4.49	8.65	0.7–30.25
	Visit 2	10	1.03	0.74	0–2.33
	Visit 3	8	0.60	0.60	0–1.9
	Visit 4	11	1.72	2.58	0.17–8.87
Inductive generalization	Visit 1	11	11.36	6.38	2.5–20.5
	Visit 2	11	8.36	4.35	3–18.5
	Visit 3	9	6.56	3.72	2–13
	Visit 4	11	11.05	8.50	2.5–28.5
Efficiency of exploration	Visit 1	11	0.12	0.08	0.03–0.29
	Visit 2	10	0.04	0.03	0.02–0.1
	Visit 3	8	0.03	0.02	0.02–0.08
	Visit 4	12	0.03	0.01	0.02–0.07
Face preferences	Visit 1	11	0.61	0.29	0–1
	Visit 2	10	0.42	0.38	0–1
	Visit 3	9	0.52	0.29	0–1
	Visit 4	11	0.36	0.31	0–1
Imitative learning	Visit 1	11	0.64	0.32	0–1
	Visit 2	12	0.80	0.26	0.5–1
	Visit 3	9	0.89	0.22	0.5–1
	Visit 4	11	1	0	–

*Visits occurred at 3-month-intervals across Phase 1: infants were 18, 21, 24, and 27 months of age for Visits 1-4, respectively*.

**Table 7:** Descriptive statistics for the Shorter- and Longer-term measures.

	**# of Participants**	***M***	***SD***	**Range: Minimum–maximum**
**SHORTER-TERM MEASURES**				
MCDI Vocabulary % rank	112	45.78	31.18	1–99
Delay of gratification (s)	103	16.66	9.65	0.33–34
**LONGER-TERM MEASURES**				
WPPSI Score (Full-scale IQ)	36	120.11	11.92	94-142
Delay of gratification (min)	33	5.16	4.39	0–15
Social communication questionnaire score	35	5.2	3.20	0–12

*Shorter-term assessments were conducted at Visit 4, after participants completed the exploratory play assessment. Longer-term assessments were conducted when children were 3 years of age*.

Additional preliminary analyses revealed no significant impact of gender, parent socioeconomic status, or testing location on children's performance on the exploratory play assessment, the shorter-term cognitive development, or the Phase 2 cognitive development measures. Thus, we collapsed across and did not consider these factors in all subsequent analyses.

#### Phase 1 analyses

We conducted three separate analyses in Phase 1. First, we looked at the items in the exploratory play assessment to determine their independence from one another and their stability across testing sessions. Second, we looked at whether the sample of infants recruited from the early intervention sites performed differently than the infants not at-risk for developmental delay on any particular exploratory play assessment item. Finally, we conducted exploratory analyses looking at the relation between the five measures in the exploratory play assessment and the shorter-term cognitive development assessment.

##### The exploratory play assessment

Our first set of analyses focused on infants' performance on the exploratory play assessment. Analyses revealed that, as intended, the exploratory play assessment tapped distinct components of exploratory play and that only performance on the efficiency measure was stable across development. This conclusion was supported by three sets of analyses. First, we conducted pairwise correlations between children's scores on all Phase 1 tasks. To control for multiple comparisons across these 10 analyses, we employed a Bonferonni-correction yielding a significance threshold level of <0.005. This analysis yielded no significant correlations among the tasks (Table 8). Second, we conducted a principal components factor analysis on children's scores on each task to determine whether the data were better described by a smaller set of components. This analyses suggested that we should not collapse the five Phase 1 items onto a fewer number of components. Although the analysis yielded three components with Eigenvalues >1, a standard threshold for extracting components, an inspection of the scree plot displaying the Eigevalues across components revealed a relatively linear decrease in Eigenvalues across the factors. Each factor contributed similarly to the overall variance—ranging from 25 to 15%—suggesting that we should retain independently all five measures in subsequent analyses. Finally, we assessed whether infants' performance was consistent across the four Phase 1 visits by conducting correlational analyses within each task across Phase 1; we applied a Bonferroni-correction for multiple comparisons within the analysis for each task, yielding a significant threshold of <0.008. This analysis revealed that only the Efficiency task was relatively stable across visits (r between.25 and.39 across four of six comparisons; see Table 9). Children did not exhibit consistent patterns of play across visits on other tasks in the exploratory play assessment.

**Table 8:** Summary of intercorrelations for the Phase 1 Exploratory Play tasks.

	**Attention to novelty**	**Inductive generalization**	**Efficiency of exploration**	**Face preference**	**Imitative learning**
Attention to novelty	–	0.03	−0.01	0.05	0.22
Inductive generalization	–	–	−0.09	0.11	0.07
Efficiency of exploration	–	–	–	0.05	−0.04
Face preference	–	–	–	–	−0.01
Imitative learning	–	–	–	–	–

*Pearson correlation r-values. N = 130 for all correlations. No correlations are significant after Bonferroni-correcting for multiple comparisons*.

**Table 9:** Stability of the Efficiency of exploration task across Phase 1.

	**Visit 1**	**Visit 2**	**Visit 3**	**Visit 4**
Visit 1	–	0.16 (*n* = 104)	0.29^*^ (*n* = 103)	0.29^*^ (*n* = 117)
Visit 2	–	–	0.39^*^ (*n* = 84)	0.08 (*n* = 95)
Visit 3	–	–	–	0.25 (*n* = 94)
Visit 4	–	–	–	–

*Pearson correlation r-values (and n for each comparison)*.

* *p < 0.008 after Bonferonni-correcting for multiple comparisons*.

##### Risk status of infants

Next, we assessed whether infants recruited from the early intervention sites differed from the infants not at-risk on any items on the exploratory play assessment. To control for multiple comparisons across the five assessment items, we employed a Bonferonni-correction yielding a threshold level of *p* < 0.01. Only the average Efficiency score differed significantly between the two populations. Independent samples *t*-tests revealed that at-risk infants were less efficient than typically-developing infants [Efficiency: typically-developing: *M* = 0.10, *SD* = 0.71, at-risk: *M* = −0.26, *SD* = 0.54, *t*_(128)_ = 2.72, *p* = 0.007, two-tailed]. There were no significant differences between typically-developing and at-risk infants on any other task in the Exploratory Play Assessment. See Table 10.

**Table 10:** Mean performance in the Phase 1 Exploratory Play Assessment as a function of risk status.

**Exploratory play task**	**Typically-developing children**	**Children at risk for developmental delay**	
Attention to novelty	−0.04(0.63)	−0.02(0.71)	*t*_(128)_ = −0.17, *p = n.s*.
Inductive generalization	0.00(0.60)	0.02(0.63)	*t*_(128)_ = −0.17, *p = n.s*.
Efficiency of exploration	0.10(0.71)	−0.26(0.54)	*t*_(128)_ = 2.72, *p* = 0.007
Face preference	52.47%(15.91%)	49.71%(16.37%)	*t*_(128)_ = 0.86, *p = n.s*.
Imitative learning	0.05(0.50)	−0.19(0.62)	*t*_(128)_ = 2.24, *p* = *n.s*.

*Mean (and Standard Deviation) for each Exploratory Play Assessment Task for the typically-developing children and children at risk for developmental delay. Note that z-scores, standardized relative to age-binned cohorts including both infants at risk and not at risk, were used for the Attention to Novelty, Inductive Generalization, Efficiency of Exploration, and Imitative Learning tasks, as they were all correlated with age. We used the raw % scores for the Social Preference task since the children's performance was not correlated with age*.

##### Shorter-term cognitive development

To motivate the hypotheses for Phase 2, we performed an exploratory analysis on the relation between each Phase 1 measure and the shorter-term cognitive development measures. As this was an exploratory analysis to motivate hypothesis-testing for Phase 2 of the study, we did not correct for multiple comparisons in this analysis. Although children produced a wide range of scores for both the MCDI and the delay of gratification tasks, the scores for both tasks were not normally distributed. Therefore, we used non-parametric Spearman rank order correlations to conduct our analyses. The only significant relation between the exploratory play tasks and the shorter-term cognitive development assessment measures was between infants' average Efficiency score and their MCDI score [*rs*_(111)_ = 0.23, *p* = 0.012; Table 11]. This correlation suggests that infants who explored more efficiently had larger vocabularies. Infants' efficiency score did not correlate with executive function abilities and did not distinguish parents with and without concerns about their child's development; similarly, no other exploratory play assessment measure predicted any other shorter-term cognitive development assessment measure.

**Table 11:** Relation between the Exploratory Play Assessment tasks and the Phase 1 Shorter-term Developmental Assessment.

**Exploratory play task**	**Vocabulary size^a^**	**Delay of gratification^a^**	**Parental concern for child's development^b^**
Attention to novelty	0.02	−0.09	*t*_(120)_ = −0.99, *p = n.s*.
Inductive generalization	0.10	0.00	*t*_(120)_ = −0.47, *p = n.s*.
Efficiency of exploration	0.23^*^	0.06	*t*_(120)_ = 0.40, *p = n.s*
Face preference	0.05	−0.06	*t*_(120)_ = 0.59, *p = n.s*
Imitative learning	0.10	0.08	*t*_(120)_ = 0.71, *p = n.s*

* *p < 0.05*.

a *Spearman's rank order correlation r-values*.

b *Independent-samples t-tests*.

#### Phase 2 analyses

A subset of children from Phase 1 (38 of 130 infants) returned for Phase 2 at 3 years of age (mean age at Phase 2 assessment: 3.23 years, *SD* = 0.15 years; range 36–43 months). Preliminary analyses revealed that this subset of children was representative of the initial sample; children who returned for Phase 2 did not differ significantly from those who did not return on either average Efficiency scores or Phase 1 vocabulary scores [Efficiency scores: Returners: *M* = 0.05, *SD* = 0.67, non-returners: *M* = −0.01, *SD* = 0.69, *t*_(128)_ = 0.45, *p* = n.s., two-tailed; Vocabulary scores: Returners: *M* = 51.12, *SD* = 28.02, non-returners: *M* = 48.21, *SD* = 31.87, *t*_(111)_ = 0.46, *p* = n.s., two-tailed].

Preliminary inspection of our longer-term developmental measures showed children's IQ scores were high (*M*: 120.1, *SD* = 11.92; range 94–142) and that no child received an SCQ score above the standard diagnostic cutoff point (i.e., 15); three children received an SCQ scores of 12, which is still below the lower cutoff point recommended for younger populations (i.e., 13; Snow and Lecavalier, 2008). This finding suggests that our sample was comprised of children with relatively high cognitive and social communication abilities, a point which we return to in the general discussion. Nonetheless, early exploratory play abilities could be related to longer-term development even among this relatively high achieving sample.

Given that infants' Efficiency score elicited the most stable performance across Phase 1, was the only measure for which typically-developing infants exhibited significant performance differences compared to the at-risk infants, and suggested a correlation with vocabulary size, we focused our final analyses only on the relation between the efficiency of children's exploration and longer-term cognitive development. Specifically, we hypothesized that greater efficiency of children's exploration in infancy would be related to higher IQ scores during Phase 2; given this specific prediction, we did not correct for multiple comparisons through the analysis of Phase 2 measures.

**Figure 4:**
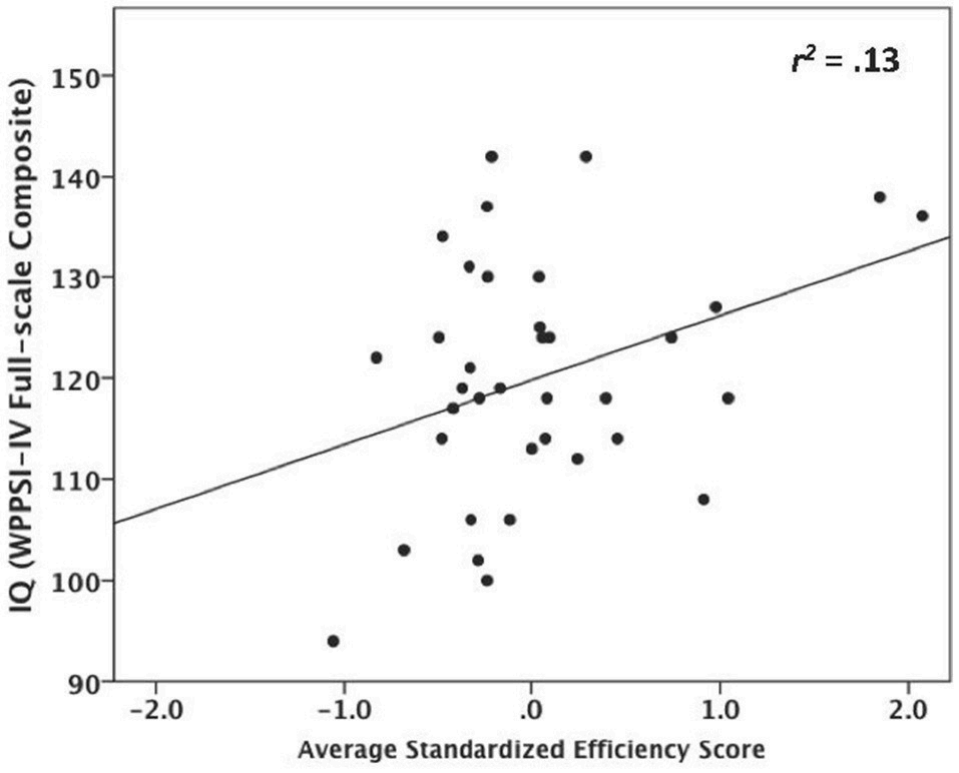
Relation between the Efficiency of exploration scores in infancy and IQ at age three.

Our analyses supported our prediction. Infants who contacted more parts of the toy relative to the time that they played had higher IQ scores at age three [*r*_(34)_ = 0.37, *p* = 0.028]; *r*^2^-values suggest a medium effect size (Figure 4). Further analysis focused specifically on individual components of IQ revealed that infants' average efficiency score was correlated significantly with verbal comprehension on the WPPSI [*r*_(34)_ = 0.35, *p* = 0.038], was marginally correlated with visual spatial skills [*r*_(34)_ = 0.28, *p* = 0.094], but not with working memory abilities [*r*_(34)_ = −0.02, *p* = 0.895]. Infants' average Efficiency score across Phase 1 did not predict children's delay of gratification or SCQ scores (both *p*s > 0.05). *Post-hoc* analyses found that no other item in the Phase 1 exploratory play assessment predicted IQ, delay of gratification, or SCQ scores (all *p*s > 0.05); additionally Phase 1 MCDI scores did not predict Phase 2 IQ scores (*p* > 0.05).

To determine whether these results held even for the youngest infants assessed, we looked at the correlation between infants' Phase 1 Visit 1 scores and all cognitive development measures for both Phase 1 and Phase 2. Analyses revealed that infants with higher Efficiency scores at their very first visit had marginally higher MCDI scores at the end of Phase 1 [Visit 1 score: *r*_(110)_ = 0.17, *p* = 0.08]. The first visit Efficiency score was also higher for typically-developing infants than at-risk infants [typically-developing: *M* = 0.14, *SD* = 1.04, at-risk: *M* = −0.41, *SD* = 0.66, *t*_(126)_ = 2.89, *p* = 0.005, two-tailed]. Finally, infants' first visit Efficiency score predicted their full-scale IQ at age three [*r*_(34)_ = 0.43, *p* = 0.009]. Further analysis revealed that infants' efficiency score was correlated significantly with verbal comprehension skills [*r*_(34)_ = 0.38, *p* = 0.021] and visual spatial skills [*r*_(34)_ = 0.39, *p* = 0.02], but not with working memory abilities [*r*_(34)_ = −0.03, *p* = 0.876]. No other Visit 1 measure predicted any cognitive development measure (all *p*s > 0.05).

## Discussion

The current study assessed the relation between and stability of multiple aspects of infants' exploratory play in a longitudinal design, as well as their relation to longer-term cognitive development. The results of the current study suggest that there are distinct, non-overlapping aspects of infants' exploratory play, and that the efficiency of infants' exploration is a relatively stable measure, at least over a 9-month period in infancy. This efficiency measure is also informative: typically developing infants' performance differed from infants at-risk for developmental delays, the measure correlates with parental report of toddlers' vocabulary, and the measure was correlated with IQ at age three. Finally, the efficiency measure appears to be related specifically to IQ: it was not correlated with children's executive function at either time point, nor did it correlate with children's social-communicative competence. In sum, a 5-min assessment of infants' free play showed that infants who explore efficiently at one time point are likely to do so again, and that the efficiency of their exploration is correlated with both near- and longer-term cognitive development.

There are several limitations to the conclusions we can draw from this study. First, we are unable to make any strong claims about the exploratory play behaviors measured in the current study—attention to novelty, inductive generalizations, face preference, and imitative learning—which were not stable over the 9-month period in Phase 1 and did not correlate with any shorter- or longer-term cognitive development measure. Critically, failure to find stable effects should not be taken to imply either that the abilities these measures were intended to index are unstable, or that those abilities have no implications for long-term cognitive development. We restricted ourselves to tasks that were easy both to administer and code. A consequence of this practical design aim may be that the simplicity of our measures limited our ability to capture relatively fine-grained individual differences in these tasks or their relation to longer-term measures of cognitive development.

In particular, we note that at least one other study has found that latency to respond to a novel vs. a familiar toy distinguishes premature infants and full-term infants (Sigman, 1976). Why did we fail to find evidence for this in our study? There are a number of possibilities. In addition to methodological variations between the studies (e.g., differences in the specific stimuli used), the care provided to premature infants has changed dramatically over the past few decades thus the behavioral profiles of premature infants in the 1970's may be different than they are today. Additionally, previous research looked at infants at a single time point (8 months) whereas the current study recruited infants from 5 to 19 months, assessed them at four different time points, and looked at infants' average score across all the tasks. Measures that are predictive at a single point in time may not be predictive averaged across 9 months of infancy. Although we did assess the relation between exploratory play at the first Phase 1 visit with longer-term developmental outcomes, this analysis included the full age range recruited for the study, rather than only young infants. Finally, it is possible that the stability of some exploratory play constructs (e.g., attention to novelty) may be captured more clearly not by assessing the relation between a uniform measurement across development (e.g., time to contact a novel toy), but rather by assessing the relation between age-calibrated measurements which may change in complexity with age (e.g., looking time measures in early infancy with action-based measures in toddlerhood).

It is also possible that, although our attention to novelty measure was intended to be comparable to visual attention measures of novelty preference, our efficiency measure may have better indexed infants' ability to process information efficiently and detect changes in their environment. As our efficiency measure was computed based on the number of parts of the toy that children contacted over their total playtime, infants with higher scores in this task may have been better able to visually detect, process, and encode novel aspects of the toy. Thus, the findings we report here may serve as supporting evidence for the positive relation between these skills and later cognitive development and suggest that the efficiency of children's manual exploration might be a proxy for measuring intelligence early in development. Future research could directly compare rate of habituation measures with our efficiency of exploration measure to determine whether they index the same cognitive abilities and whether they are related similarly to cognitive development.

Our design also is unable to assess the full complexity of the development of children's exploratory play. In particular, as noted in the introduction, studies have shown that infants' manual exploration becomes more complex and integrated with other cognitive processes over development. As children's motor repertoire increases over development, children are able to engage simultaneously with more objects, both exploring interactions between these objects and using objects as tools to explore their environment, which can facilitate the acquisition and learning of new knowledge (e.g., Lockman, 2000). Future research could be directed at assessing behaviors across the full range of contexts and actions that define children's developing exploratory play, ranging from simple exploration of single objects to the use of multi-affordance objects as tools. Moreover, given that children's exploratory play behaviors were standardized according to age-matched peers to reduce age effects over our sample, the findings from this study motivate future research with larger samples that could investigate the time-course of developmental changes within components of exploratory play at both at the level of individual children and within smaller developmental windows, how developmental changes compare across components of exploratory play, and how they collectively interact to impact cognitive development outcomes.

The current results are also limited in that the children retained through Phase 2 had relatively high IQ scores (*M*: 120.1, *SD* = 11.9; range 94–142). We do not know whether the correlation between exploratory play and IQ holds for the broader population–nor do we now whether infants' exploratory play, even in relatively high IQ children, predicts intelligence after age three. Additionally, future research might look at whether children's home environment plays a mediating or moderating role in the relation between exploratory play and cognitive development (e.g., having more toys in the home may independently facilitate children's exploration and their later cognitive development or the relation between exploration and cognitive development may only hold for homes with many toys to explore) (e.g., see Storch and Whitehurst, 2001, for similar approach in literacy development). Finally, this study leaves unresolved the question of causation; smarter infants might explore more efficiently or efficient exploration might contribute to intelligence. Future research might identify the particular processes underlying the correlation between efficient exploratory play and intelligence.

Despite these limitations our results suggest a positive relation between the efficiency of exploratory play and cognitive development. There are several possible mechanisms that might contribute to this correlation. Although our exploratory play assessment was designed to involve comparable motor demands across tasks (reaching for and manipulating objects), and although infants did not differ on other measures of motor capability (e.g., latency to reach for novel objects) it is nonetheless possible that infants who discovered more functions of a toy relative to their total play-time had more advanced motor skills overall (see e.g., Bornstein et al., 2013). If so, it may be that infants who are relatively advanced in their motor development are relatively advanced in cognitive development as well, that advances in motor development contribute to cognitive development through enhanced opportunities for interaction and exploration, or that exploratory play has differential effects on children at varying stages of motor development (e.g., Bushnell and Boudreau, 1993; Karasik et al., 2011; Schwarzer et al., 2013; Kretch et al., 2014) However, assuming that differences in infants' motor skills are not the only factor affecting the efficiency of their exploratory play, the free exploration measure may have taxed a number of other cognitive abilities. Efficient exploration plausibly requires the ability to flexibly engage and disengage attention, to plan sequences of actions, and to integrate these abilities with sensitivity to the rate of information gain. Arguably, the cognitive skills that let infants rapidly discover novel functions of a toy could be deployed to support learning in many domains. Finally, it is possible that motivational factors underlie both children's performance on the efficiency measure and their performance on the cognitive measures. Future research might clarify the relative contribution of motor skills, cognitive abilities, and affective engagement to the correlation between efficient exploratory play and later cognitive developments. Additionally, although we found evidence of a specific relation between efficient exploration and verbal abilities, future research might study more broadly the relation between efficient exploration and different components of IQ (i.e., verbal and spatial abilities) and of executive function (e.g., inhibition, set shifting, working memory) across development.

The current study suggests that continued research investigating individual differences in early exploration may have important implications for our understanding of longer-term cognitive developments. It is also encouraging that stable, predictive differences in infants' exploratory play can be assessed using stimuli and measures easy to administer outside of the lab. Such measures have the potential to link basic science on children's exploratory play with applied efforts to identify children at-risk, and intervene on children's cognitive development. Insofar as infants' free exploration predicts longer-term cognitive development, children's play is worth taking seriously.

## Author contributions

PM and LS designed the study. PM oversaw data collection for the study. EH was responsible for Phase 1 data collection. PM and EH oversaw coding of all data. PM, EH, and LS all contributed to data analysis and interpretation, and PM, EH, and LS all contributed to the drafting and revision of the manuscript.

### Conflict of interest statement

The authors declare that the research was conducted in the absence of any commercial or financial relationships that could be construed as a potential conflict of interest.

## References

[B1] Allen C. W. Silove N. Williams K. Hutchins P. (2007). Validity of the social communication questionnaire in assessing risk of autism in preschool children with developmental problems. J. Autism Dev. Disord. 37, 1272–1278. 10.1007/s10803-006-0279-7 17080270

[B2] American Psychiatric Association (2013). Diagnostic and Statistical Manual of Mental Disorders (DSM-5®). Arlington, VA: American Psychiatric Publications.

[B3] Astington J. Jenkins J. (1995). Theory of mind development and social understanding. Cogn. Emot. 9, 151–165. 10.1080/02699939508409006

[B4] Baldwin D. A. Markman E. M. Melartin R. L. (1993). Infants' ability to draw inferences about nonobvious object properties: evidence from exploratory play. Child Dev. 64, 711–728. 10.2307/1131213 8339691

[B5] Baumgartner H. Oakes L. (2013). Investigating the relation between infants' manual activity with objects and their perception of dynamic events. Infancy 18, 983–1006. 10.1111/infa.12009

[B6] Berlyne D. (1969). Laughter, humor, and play. Handb. Soc. Psychol. 3, 795–852.

[B7] Bjorklund D. (1997). The role of immaturity in human development. Psychol. Bull. 122, 153–169. 10.1037/0033-2909.122.2.153 9283298

[B8] Bjorklund D. F. Brown R. D. (1998). Physical play and cognitive development: integrating activity, cognition, and education. Child Dev. 69, 604–606. 10.1111/j.1467-8624.1998.tb06229.x 9680673

[B9] Bonawitz E. Shafto P. Gweon H. Goodman N. D. Spelke E. Schulz L. (2011). The double-edged sword of pedagogy: instruction limits spontaneous exploration and discovery. Cognition 120, 322–330. 10.1016/j.cognition.2010.10.001 21216395 PMC3369499

[B10] Bonawitz E. B. van Schijndel T. J. Friel D. Schulz L. (2012). Children balance theories and evidence in exploration, explanation, and learning. Cogn. Psychol. 64, 215–234. 10.1016/j.cogpsych.2011.12.002 22365179

[B11] Bornstein M. (1985). How infant and mother jointly contribute to developing cognitive competence in the child. Proc. Natl. Acad. Sci. U.S.A. 82, 7470–7473. 10.1073/pnas.82.21.7470 3864165 PMC391367

[B12] Bornstein M. H. Hahn C. S. Suwalsky J. T. (2013). Physically developed and exploratory young infants contribute to their own long-term academic achievement. Psychol. Sci. 24, 1906–1917. 10.1177/0956797613479974 23964000 PMC4151610

[B13] Bornstein M. H. Benasich A. A. (1986). Infant habituation: assessments of individual differences and short-term reliability at five months. Child Dev. 57, 87–99. 10.2307/1130640 3948596

[B14] Bourgeois K. S. Khawar A. W. Neal S. A. Lockman J. J. (2005). Infant manual exploration of objects, surfaces, and their interrelations. Infancy 8, 233–252. 10.1207/s15327078in0803_3

[B15] Bruner J. Jolly A. Sylva K. (1976). Play: It's Role in Development and Evolution (New York, NY: Basic Books).

[B16] Buchsbaum D. Bridgers S. Weisberg D. Gopnik A. (2012). The power of possibility: causal learning, counterfactual reasoning, and pretend play. Philos. Trans. R. Soc. Lon. B Biol. Sci. 367, 2202–2212. 10.1098/rstb.2012.0122 22734063 PMC3385687

[B17] Bushnell E. W. Boudreau J. P. (1993). Motor development and the mind: the potential role of motor abilities as a determinant of aspects of perceptual development. Child Dev. 64, 1005–1021. 10.2307/1131323 8404253

[B18] Butler L. P. Markman E. M. (2014). Preschoolers use pedagogical cues to guide radical reorganization of category knowledge. Cognition 130, 116–127. 10.1016/j.cognition.2013.10.002 24211439

[B19] Butler L. P. Tomasello M. (2016). Two-and 3-year-olds integrate linguistic and pedagogical cues in guiding inductive generalization and exploration. J. Exp. Child Psychol. 145, 64–78. 10.1016/j.jecp.2015.12.001 26826468

[B20] Carey S. Zaitchik D. Bascandziev I. (2015). Theories of development: in dialog with Jean Piaget. Dev. Rev. 38, 36–54. 10.1016/j.dr.2015.07.003

[B21] Carlson S. M. Moses L. J. (2001). Individual differences in inhibitory control and children's theory of mind. Child Dev. 72, 1032–1053. 10.1111/1467-8624.00333 11480933

[B22] Cassia V. M. Simion F. (2002). Individual differences in object-examining duration: do they reflect the use of different encoding strategies? Cogn. Dev. 17, 1219–1234. 10.1016/S0885-2014(02)00113-2

[B23] Colombo J. Mitchell D. W. Horowitz F. D. (1988). Infant visual attention in the paired-comparison paradigm: test-retest and attention-performance relations. Child Dev. 59, 1198–1210. 10.2307/1130483 3168636

[B24] Colombo J. Mitchell D. W. O'Brien M. Horowitz F. (1987). The stability of visual habituation during the first year of life. Child Dev. 58, 474–487. 10.2307/1130524 3829788

[B25] Colombo J. Shaddy D. Richman W. Maikranz J. Blaga O. (2004). The developmental course of habituation in infancy and preschool outcome. Infancy 5, 1–38. 10.1207/s15327078in0501_1

[B26] Cook C. Goodman N. Schulz L. (2011). Where science starts: Spontaneous experiments in preschoolers' exploratory play. Cognition 120, 341–349. 10.1016/j.cognition.2011.03.003 21561605

[B27] Csibra G. Gergely G. (2006). Social learning and social cognition: the case for pedagogy, in Processes of Change in Brain and Cognitive Development. Attention and Performance, eds Munakata Y. Johnson M. H. (Oxford: Oxford University Press), 249–274.

[B28] de Almeida Soares D. von Hofsten C. Tudella E. (2012). Development of exploratory behavior in late preterm infants. Infant Behav. Dev. 35, 912–915. 10.1016/j.infbeh.2012.09.002 23069127

[B29] de Campos A. C. da Costa C. S. Savelsbergh G. J. Rocha N. A. (2013). Infants with Down syndrome and their interactions with objects: development of exploratory actions after reaching onset. Res. Dev. Disabil. 34, 1906–1916. 10.1016/j.ridd.2013.03.001 23584171

[B30] Dewar K. M. Xu F. (2010). Induction, overhypothesis, and the origin of abstract knowledge: evidence from 9-month-old infants. Psychol. Sci. 21, 1871–1877. 10.1177/0956797610388810 21078899

[B31] Fagan J. F. (1974). Infant recognition memory: the effects of length of familiarization and type of discrimination task. Child Dev. 45, 351–356. 10.1111/j.1467-8624.1974.tb00603.x 4837713

[B32] Fagan J. F. Holland C. R. Wheeler K. (2007). The prediction, from infancy, of adult IQ and achievement. Intelligence 35, 225–231. 10.1016/j.intell.2006.07.007

[B33] Fantz R. L. (1963). Pattern vision in newborn infants. Science 140, 296–297. 10.1126/science.140.3564.296 17788054

[B34] Farroni T. Csibra G. Simion F. Johnson M. H. (2002). Eye contact detection in humans from birth. Proc. Natl. Acad. Sci. U.S.A. 99, 9602–9605. 10.1073/pnas.152159999 12082186 PMC123187

[B35] Fenson L. Kagan J. Kearsley R. B. Zelazo P. R. (1976). The developmental progression of manipulative play in the first two years. Child Dev. 47, 232–236. 10.2307/1128304

[B36] Fenson L. Pethick S. Renda C. Cox J. Dale P. Reznick J. (2000). Short-form versions of the MacArthur communicative development inventories. Appl. Psycholinguist. 21, 95–116. 10.1017/S0142716400001053

[B37] Fenson L. Sapper V. Miner D. G. (1974). Attention and manipulative play in the one-year-old child. Child Dev. 45, 757–764. 10.2307/1127842 4143830

[B38] Frank M. C. Vul E. Johnson S. P. (2009). Development of infants' attention to faces during the first year. Cognition 110, 160–170. 10.1016/j.cognition.2008 19114280 PMC2663531

[B39] Frank M. C. Vul E. Saxe R. (2012). Measuring the development of social attention using free-viewing. Infancy 17, 355–375. 10.1111/j.1532-7078.2011.00086.x 32693486

[B40] Gerson S. A. Woodward A. L. (2014). The joint role of trained, untrained, and observed actions at the origins of goal recognition. Infant Behav. Dev. 37, 94–104. 10.1016/j.infbeh.2013.12.013 24468646 PMC3951724

[B41] Gopnik A. Walker C. (2013). Considering counterfactuals: the relationship between causal learning and pretend play. Am. J. Play 6, 15–28.

[B42] Gopnik A. Wellman H. M. (2012). Reconstructing constructivism: causal models, bayesian learning mechanisms, and the theory theory. Psychol. Bull. 138:1085 10.1037/a0028044 22582739 PMC3422420

[B43] Groos K. (1901). The Play of Man, (New York, NY: Appleton).

[B44] Groos K. Baldwin E. (1898). The Play of Animals, (New York, NY, Appleton).

[B45] Gweon H. Pelton H. Konopka J. A. Schulz L. E. (2014). Sins of omission: children selectively explore when teachers are under-informative. Cognition 132, 335–341. 10.1016/j.cognition.2014.04.013 24873737

[B46] Gweon H. Tenenbaum J. B. Schulz L. E. (2010). Infants consider both the sample and the sampling process in inductive generalization. Proc. Natl. Acad. Sci. U.S.A. 107, 9066–9071. 10.1073/pnas.1003095107 20435914 PMC2889113

[B47] Hutt C. Bhavani R. (1972). Predictions from play. Nature 237, 171–172. 10.1038/237171b0 4555512

[B48] Johnson D. Brody N. (1977). Visual habituation, sensorimotor development, and tempo of play in one-year-old infants. Child Dev. 48, 315–319. 10.2307/1128920 844357

[B49] Johnson M. H. (2005). Subcortical face processing. Nat. Rev. Neurosci. 6, 766–774. 10.1038/nrn1766 16276354

[B50] Johnson M. H. Senju A. Tomalski P. (2015). The two-process theory of face processing: modifications based on two decades of data from infants and adults. Neurosci. Biobehav. Rev. 50, 169–179. 10.1016/j.neubiorev.2014.10.009 25454353

[B51] Kahrs B. A. Jung W. P. Lockman J. J. (2013). Motor origins of tool use. Child Dev. 84, 810–816. 10.1111/cdev.12000 23106197 PMC3839412

[B52] Karasik L. B. Tamis-LeMonda C. S. Adolph K. E. (2011). Transition from crawling to walking and infants' actions with objects and people. Child Dev. 82, 1199–1209. 10.1111/j.1467-8624.2011.01595.x 21545581 PMC3163171

[B53] Kaur M. Srinivasan S. M. Bhat A. N. (2015). Atypical object exploration in infants at-risk for autism during the first year of lifer. Front. Psychol. 6:798. 10.3389/fpsyg.2015.00798 26136702 PMC4468838

[B54] Kavšek M. (2004). Predicting later IQ from infant visual habituation and dishabituation: a meta-analysis. J. Appl. Dev. Psychol. 25, 369–393. 10.1016/j.appdev.2004.04.006

[B55] Kavsek M. Bornstein M. H. (2010). Visual habituation and dishabituation in preterm infants: a review and meta-analysis. Res. Dev. Disabil. 31, 951–975. 10.1016/j.ridd.2010.04.016 20488657 PMC3167676

[B56] Kopp C. B. Vaughn B. E. (1982). Sustained attention during exploratory manipulation as a predictor of cognitive competence in preterm infants. Child Dev. 53, 174–182. 10.2307/1129650 7060420

[B57] Koterba E. A. Leezenbaum N. B. Iverson J. M. (2014). Object exploration at 6 and 9 months in infants with and without risk for autism. Autism 18, 97–105. 10.1177/1362361312464826 23175749 PMC3773524

[B58] Kretch K. S. Franchak J. M. Adolph K. E. (2014). Crawling and walking infants see the world differently. Child Dev. 85, 1503–1518. 10.1111/cdev.12206 24341362 PMC4059790

[B59] Legare C. (2012). Exploring explanation: explaining inconsistent evidence informs exploratory, hypothesis-testing behavior in young children. Child Dev. 83, 173–185. 10.1111/j.1467-8624.2011.01691.x 22172010

[B60] Legare C. H. (2014). The contributions of explanation and exploration to children's scientific reasoning. Child Dev. Perspect. 8, 101–106. 10.1111/cdep.12070

[B61] Leslie A. (1987). Pretense and representation: the origins of “theory of mind. Psychol. Rev. 94, 412–426. 10.1037/0033-295X.94.4.412

[B62] Libertus K. Needham A. (2010). Teach to reach: the effects of active vs. passive reaching experiences on action and perception. Vision Res. 50, 2750–2757. 10.1016/j.visres.2010.09.001 20828580 PMC2991490

[B63] Lockman J. (2000). A perception-action perspective on tool use development. Child Dev. 71, 137–144. 10.1111/1467-8624.00127 10836567

[B64] Loveland K. A. (1987). Behavior of young children with Down syndrome before the mirror: exploration. Child Dev. 58, 768–778. 10.2307/1130213 2956072

[B65] Marchman V. A. Fernald A. (2008). Speed of word recognition and vocabulary knowledge in infancy predict cognitive and language outcomes in later childhood. Dev. Sci. 11, F9–F16. 10.1111/j.1467-7687.2007.00671.x 18466367 PMC2905590

[B66] McCall R. B. (1974). Exploratory manipulation and play in the human infant. Monographs of the society for research in child development 39, 1–88. 10.2307/1166007 4444723

[B67] McCall R. B. Carriger M. S. (1993). A meta-analysis of infant habituation and recognition memory performance as predictors of later IQ. Child Dev. 64, 57–79. 10.2307/1131437 8436038

[B68] Meltzoff A. N. (2007). ‘Like me’: a foundation for social cognition. Dev. Sci. 10, 126–134. 10.1111/j.1467-7687.2007.00574.x 17181710 PMC1852489

[B69] Mischel W. Shoda Y. Rodriguez M. (1989). Delay of gratification in children. Science 244, 933–938. 10.1126/science.2658056 2658056

[B70] Morange-Majoux F. Cougnot P. Bloch H. (1997). Hand tactual exploration of textures in infants from 4 to 6 months. Infant Child Dev. 6, 127–136. 10.1002/(SICI)1099-0917(199709/12)6:3/4<127::AID-EDP152>3.0.CO;2-G

[B71] Morton J. Johnson M. H. (1991). CONSPEC and CONLERN: a two-process theory of infant face recognition. Psychol. Rev. 98, 164–181. 10.1037/0033-295X.98.2.164 2047512

[B72] Needham A. Barrett T. Peterman K. (2002). A pick-me-up for infants' exploratory skills: Early simulated experiences reaching for objects using ‘sticky mittens’ enhances young infants' object exploration skills. Infant Behav. Dev. 25, 279–295. 10.1016/S0163-6383(02)00097-8

[B73] Oakes L. M. Kannass K. N. Shaddy D. J. (2002). Developmental changes in endogenous control of attention: The role of target familiarity on infants' distraction latency. Child Dev. 73, 1644–1655. 10.1111/1467-8624.00496 12487484

[B74] Oakes L. M. Madole K. L. Cohen L. B. (1991). Infants' object examining: habituation and categorization. Cogn. Dev. 6, 377–392. 10.1016/0885-2014(91)90045-F

[B75] Oakes L. M. Tellinghuisen D. J. (1994). Examining in infancy: does it reflect active processing? Dev. Psychol. 30:748–756. 10.1037/0012-1649.30.5.748

[B76] Oudgenoeg-Paz O. Leseman P. P. Volman M. C. (2015). Exploration as a mediator of the relation between the attainment of motor milestones and the development of spatial cognition and spatial language. Dev. Psychol. 51, 1241–1253. 10.1037/a0039572 26192037

[B77] Palmer C. F. (1989). The discriminating nature of infants' exploratory actions. Dev. Psychol. 25, 885–893. 10.1037/0012-1649.25.6.885

[B78] Pellegrini A. Dupuis D. Smith P. (2007). Play in evolution and development. Dev. Rev. 27, 261–276. 10.1016/j.dr.2006.09.001

[B79] Pellegrini A. D. Smith P. K. (1998). Physical activity play: the nature and function of a neglected aspect of play. Child Dev. 69, 577–598. 10.1111/j.1467-8624.1998.tb06226.x 9680672

[B80] Perone S. Oakes L. M. (2006). It Clicks When It Is Rolled and It Squeaks When It Is Squeezed: what 10-month-old infants learn about object function. Child Dev. 77, 1608–1622. 10.1111/j.1467-8624.2006.00962.x 17107449

[B81] Piaget J. (1962). Play, Dreams and Imitation in Childhood, (New York, NY: Norton).

[B82] Powell L. J. Carey S. (2017). Executive function depletion in children and its impact on theory of mind. Cognition 164, 150–162. 10.1016/j.cognition.2017.03.022 28427031

[B83] Power T. (2000). Play and Exploration in Children and Animals, (Hillsdale, NJ Lawrence-Erlbaum).

[B84] Raine A. Reynolds C. Venables P. H. Mednick S. A. (2002). Stimulation seeking and intelligence: a prospective longitudinal study. J. Pers. Soc. Psychol. 82, 663–674. 10.1037//0022-3514.82.4.663 11999930

[B85] Rakison D. H. Krogh L. (2012). Does causal action facilitate causal perception in infants younger than 6 months of age? Dev. Sci. 15, 43–53. 10.1111/j.1467-7687.2011.01096.x 22251291

[B86] Reid V. M. Dunn K. Young R. J. Amu J. Donovan T. Reissland N. (2017). The human fetus preferentially engages with face-like visual stimuli. Curr. Biol. 27, 1825–1828. 10.1016/j.cub.2017.05.044 28602654

[B87] Richards J. E. (1997). Effects of attention on infants' preference for briefly exposed visual stimuli in the paired-comparison recognition-memory paradigm. Dev. Psychol. 33:22 10.1037/0012-1649.33.1.22 9050387

[B88] Rochat P. (1989). Object manipulation and exploration in 2-to 5-month-old infants. Dev. Psychol. 25, 871–884. 10.1037/0012-1649.25.6.871

[B89] Rose S. A. (1983). Differential rates of visual information processing in full-term and preterm infants. Child Dev. 54, 1189–1198. 10.2307/1129674 6354626

[B90] Rose S. A. Feldman J. F. (1987). Infant visual attention: stability of individual differences from 6 to 8 months. Dev. Psychol. 23:490 10.1037/0012-1649.23.4.490

[B91] Rose S. A. Feldman J. F. Jankowski J. J. (2001). Attention and recognition memory in the 1st year of life: a longitudinal study of preterm and full-term infants. Dev. Psychol. 37, 135–151. 10.1037/0012-1649.37.1.135 11206428

[B92] Rose S. A. Gottfried A. W. Melloy-Carminar P. Bridger W. H. (1982). Familiarity and novelty preferences in infant recognition memory: implications for information processing. Dev. Psychol. 18, 704–713. 10.1037/0012-1649.18.5.704

[B93] Rubin K. Fein G. Vandenberg B. (1983). Play, in The Handbook of Child Psychology, ed Hetherington E. M. (New York, NY: Wiley), 693–774.

[B94] Ruff H. A. (1984). Infants' manipulative exploration of objects: effects of age and object characteristics. Dev. Psychol. 20:9 10.1037/0012-1649.20.1.9

[B95] Ruff H. A. (1986). Components of attention during infants' manipulative exploration. Child Dev. 57, 105–114. 10.2307/1130642 3948587

[B96] Ruff H. A. Dubiner K. (1987). Stability of individual differences in infants' manipulation and exploration of objects. Percept. Mot. Skills 64, 1095–1101. 10.2466/pms.1987.64.3c.1095 3627912

[B97] Ruff H. A. Saltarelli L. M. Capozzoli M. Dubiner K. (1992). The differentiation of activity in infants' exploration of objects. Dev. Psychol. 28, 851–861. 10.1037/0012-1649.28.5.851

[B98] Ruff H. A. McCarton C. Kurtzberg D. Vaughn H. G. (1984). Preterm infants' manipulative exploration of objects. Child Dev. 55, 1166–1173. 6488951

[B99] Rutter M. Bailey A. Berument S. Lord C. Pickles A. (2003). Social Communication Questionnaire. Torrance, CA: Western Psychological Services.

[B100] Schulz L. (2012). The origins of inquiry: inductive inference and exploration in early childhood. Trends Cogn. Sci. 16, 382–389. 10.1016/j.tics.2012.06.004 22721579

[B101] Schulz L. E. Bonawitz E. B. (2007). Serious fun: preschoolers engage in more exploratory play when evidence is confounded. Dev. Psychol. 43, 1045–1050. 10.1037/0012-1649.43.4.1045 17605535

[B102] Schulz L. Standing H. Bonawitz E. B. (2008). Word, thought and deed: the role of object labels in children's inductive inferences and exploratory play. Dev. Psychol. 44, 1266–1276. 10.1037/0012-1649.44.5.1266 18793061

[B103] Schwarzer G. Frietag C. Schum N. (2013). How crawling and manual object exploration are related to the mental rotation abilities of 9-month-old infants. Front. Psychol. 4:97. 10.3389/fpsyg.2013.00097 23459565 PMC3586719

[B104] Senju A. Csibra G. (2008). Gaze following in human infants depends on communicative signals. Curr. Biol. 18, 668–671. 10.1016/j.cub.2008.03.059 18439827

[B105] Shneidman L. Gweon H. Schulz L. E. Woodward A. L. (2016). Learning from others and spontaneous exploration: a cross-cultural investigation. Child Dev. 87, 723–735. 10.1111/cdev.12502 27189400

[B106] Shoda Y. Mischel W. Peake P. K. (1990). Predicting adolescent cognitive and social competence from preschool delay of gratification: identifying diagnostic conditions. Dev. Psychol. 26, 978–986. 10.1037/0012-1649.26.6.978

[B107] Sigman M. (1976). Early development of preterm and full-term infants: exploratory behavior in eight-month-olds.Child Dev. 47, 606–612.

[B108] Sim Z. L. Xu F. (2017). Learning higher-order generalizations through free play: evidence from 2-and 3-year-old children. Dev. Psychol. 53, 642–651. 10.1037/dev0000278 28333526

[B109] Singer D. Golinkoff R. Hirsh-Pasek K. (2006). Play = *Learning: How Play Motivates and Enhances Children's Cognitive and Social-Emotional Growth*, (New York, NY: Oxford University Press).

[B110] Snow A. V. Lecavalier L. (2008). Sensitivity and specificity of the modified checklist for autism in toddlers and the social communication questionnaire in preschoolers suspected of having pervasive developmental disorders. Autism 12, 627–644. 10.1177/1362361308097116 19005032

[B111] Sommerville J. A. Woodward A. L. Needham A. (2005). Action experience alters 3-month-old infants' perception of others' actions. Cognition 96, B1–B11. 10.1016/j.cognition.2004.07.004 15833301 PMC3908452

[B112] Soska K. C. Adolph K. E. Johnson S. P. (2010). Systems in development: motor skill acquisition facilitates three-dimensional object completion. Dev. Psychol. 46, 129–138. 10.1037/a0014618 20053012 PMC2805173

[B113] Southgate V. Chevallier C. Csibra G. (2009). Sensitivity to communicative relevance tells young children what to imitate. Dev. Sci. 12, 1013–1019. 10.1111/j.1467-7687.2009.00861.x 19840055

[B114] Stahl A. E. Feigenson L. (2015). Observing the unexpected enhances infants' learning and exploration. Science 348, 91–94. 10.1126/science.aaa3799 25838378 PMC5861377

[B115] Storch S. A. Whitehurst G. J. (2001). The role of family and home in the literacy development of children from low-income backgrounds. New Dir. Child Adolesc. Dev. 53–72. 10.1002/cd.15 11468866

[B116] Taylor M. Carlson S. (1997). The relation between individual differences in fantasy and theory of mind. Child Dev. 68, 436–455. 10.2307/1131670 9249959

[B117] Téglás E. Vul E. Girotto V. Gonzalez M. Tenenbaum J. B. Bonatti L. L. (2011). Pure reasoning in 12-month-old infants as probabilistic inference. Science 332, 1054–1059. 10.1126/science.1196404 21617069

[B118] Tenenbaum J. B. Kemp C. Griffiths T. L. Goodman N. D. (2011). How to grow a mind: statistics, structure, and abstraction. Science 331, 1279–1285. 10.1126/science.1192788 21393536

[B119] Tomasello M. (2000). Culture and cognitive development. Curr. Dir. Psychol. Sci. 9, 37–40. 10.1111/1467-8721.00056

[B120] van Schijndel T. Visser J. van Bers B. Rajimakers M. (2015). Preschoolers perform more informative experiments after observing theory-violating evidence. J. Exp. Child Psychol. 131, 104–119. 10.1016/j.jecp.2014.11.008 25544394

[B121] Viholainen H. Ahonen T. Lyytinen P. Cantell M. Tolvanen A. Lyytinen H. (2006). Early motor development and later language and reading skills in children at risk of familial dyslexia. Dev. Med. Child Neurol. 48, 367–373. 10.1017/S001216220600079X 16608545

[B122] Vygotsky L. (1934/1962). Thought and Language. Trans. E. Hanfmann and G. Vakar. Cambridge: MIT Press.

[B123] Wechsler D. (2012). Wechsler Preschool and Primary Scale of Intelligence-4th Edn., Technical and Interpretative Manual. (San Antonio, TX: Psychological Corp).

[B124] Welder A. N. Graham S. A. (2001). The influence of shape similarity and shared labels on infants' inductive inferences about nonobvious object properties. Child Dev. 72, 1653–1673. 10.1111/1467-8624.00371 11768138

[B125] Whyte V. A. McDonald P. V. Baillargeon R. Newell K. M. (1994). Mouthing and grasping of objects by young infants. Ecol. Psychol. 6, 205–218. 10.1207/s15326969eco0603_3

[B126] Xu F. Kushnir T. (2013). Infants are rational constructivist learners. Curr. Dir. Psychol. Sci. 22, 28–32. 10.1177/0963721412469396

[B127] Youngblade L. M. Dunn J. (1995). Individual differences in young children's pretend play with mother and sibling: links to relationships and understanding of other people's feelings and beliefs. Child Dev. 66, 1472–1492. 10.2307/1131658 7555225

[B128] Zuccarini M. Sansavini A. Iverson J. M. Savini S. Guarini A. Alessandroni R.. (2016). Object engagement and manipulation in extremely preterm and full term infants at 6 months of age. Res. Dev. Disabil. 55, 173–184. 10.1016/j.ridd.2016.04.001 27101093

